# A look back: Coenesthetic schizophrenia. A literature review.

**DOI:** 10.1192/j.eurpsy.2023.2303

**Published:** 2023-07-19

**Authors:** R. Galerón Guzmán, M. Huete Naval, E. Herrero Pellón, P. Albarracín Marcos, A. García Recio

**Affiliations:** Hospital Clínico San Carlos, Madrid, Spain

## Abstract

**Introduction:**

We present the case of a 19-year-old patient who experienced a nonexisting moving sensation, increasing size and painful sensation on tongue, jaw and skull bones. Likewise, the patient showed high anguish, psychomotor restlessness and low mood, in relation to somatic symptomatology; which severely interfered in his life, dropping his university studies and his social life. He also presented thoughts of being victim of a complot of his classmates.

**Objectives:**

To present a case report and to review the literature of coenesthetic schizophrenia.

**Methods:**

Literature review of scientific articles searching in EMBASE and Pubmed. We considered articles in English and Spanish.

**Results:**

Treatment with oral aripiprazole and sertraline was started, with progressive clinical improvement, decreasing somatic sensations until they disappeared, as well as mood improvement and remission of anxiety and psychomotor restlessness.

Coenesthetic schizophrenia was first described in 1957 by Gerd Huber. It is characterized by bodily sensations often combined with affective disturbances. Other symptoms that occur frequently are affective, vegetative, motor and sensory alterations. Typical schizophrenic symptoms are limited to brief psychotic episodes.

**Conclusions:**

We consider knowledge of this entity important, given the differential clinical characteristics regarding to other presentations of schizophrenia, as well as the need to do a differential diagnosis with other disorders such as body dysmorphic disorder or hypochondriasis.

**Disclosure of Interest:**

None Declared

